# Modern Sociology

**Published:** 1896-11-21

**Authors:** 


### Modern Sociology.

### MISTAKEN IMPRESSIONS OF PRISON LIFE.—III.

The recent institution of the First Offenders' Act, which is now in full working order, is a distinctly wise and beneficent innovation on the code of judicial enactments hitherto in force. Some slight misconception exists with regard to it, inasmuch as it has until lately been supposed to apply only to young criminals, and not to be in any sense available for those of mature age. A typical case, which occurred recently, has drawn forth from the able originator of the Act the statement that, although his primary object had no doubt been to inaugurate a new and improved mode of dealing with the young, he yet had not in the least intended to exclude older offenders from the benefit of the measure, and he expressed himself well pleased that the magistrate, who judged the case of a respectable man suddenly tempted to a petty theft, had acted upon it. The precise method in which this Act operates is as follows: Any person, old or young, who is brought to the bar of justice for an offence which, though undeniable, is yet the first he has ever been known to commit, may be simply reprimanded and discharged without fine or imprisonment, but with the understanding that his appearing on that occasion will hold against him with all the weight of a first conviction should he perpetrate any second offence entailing punishment, while it will remain as a dead letter, so far as he is concerned, if his conduct from that time forward is perfectly honest and straightforward. In speaking of the effect of previous convictions on the fate of a prisoner, we touch on another vexed question as to the administration of justice on which very varying opinions are held by the public. Many persons consider that a prisoner brought to trial, and found guilty of the crime for which he has to answer at the moment, ought to be punished for that special offence only, without any reference to his antecedents, whatever they may have been. It is, indeed, we believe, undoubtedly true that some of the authorities, acting in subordinate judicial positions, hold to this principle so strongly that they refuse to take any notice of former convictions at all, and sentence the offender on the ground of the misdeed actually before them, and on that alone. What this proceeding on their part practically means is very well summed up by the writer of the article on professional crime to which we have referred, and we will quote his words as to the action of these magistrates: "They are fully entitled to their opinions, but when they give effect to their opinions on the judgment seat, they set themselves against the law as flagrantly as the criminals they send to gaol, for, rightly or wrongly, the Legislature has decided, after full and careful enquiry, and in the most definite manner, that a previous conviction of crime is always to be taken into account." That the decision of the State on this point is perfectly right and wise no one who is cognisant of the practical working of the system can possibly deny. The result of ignoring previous convictions in the case, say, of two men charged with offences of a corresponding magnitude, and sentenced to the same short term of imprisonment, might very probably be as follows: One of the two, only lately fallen into evil ways, and never before actually punished by law, receives so salutary a warning by his first bitter taste of imprisonment, that he is checked finally in a nefarious career, and, with the assistance of good advice within the prison walls, and judicious help on his discharge, he enters on a course of reformation from which he never again departs. The other (like some men of whom we have personal knowledge) has been convicted again and again—ten, twenty, or even thirty times—and sentenced to terms of imprisonment varying in length, till the greater part of his mature life has been spent in gaol. He is known to the police as the leader of a notorious gang of burglars; he is of the class who in these days perform their robberies revolver in hand, and are ready to act on the proverb that dead men tell no tales whenever they find it advisable for their own safety to silence a witness of their crimes. He is a deadly peril and injury to society; the corrupter of the young, the cruel oppressor of the helpless; and when the few weeks' imprisonment to which he has been sentenced are over, he is let loose on the world again to work what evil he will. The record of his previous convictions, if duly noted and acted upon, would have inevitably gained for him five, or more probably ten years' penal servitude, during which time the community in general would be safe from the risks and evils involved in his presence amongst them when at liberty.

There are questions connected with the right treatment of professional criminals, as well as on some other cognate points, which we are anxious to bring before our readers, but for which the space remaining to us at present is too limited, so that they must be left for a future opportunity. We will, therefore, for the present employ ourselves in endeavouring to disabuse the public mind of any views respecting the punishment of first-class misdemeanants which might lead them to think with anxiety or undue regret of the sentence pronounced on Dr. Jameson and his colleagues in the Transvaal raid. With the exception of the unfortunate officer whose severe wounds caused him, we fear, as much suffering in the well-appointed homes of his relatives as in Holloway Prison, it is quite unnecessary to expend any serious compassion on the gentlemen condemned to remain for longer or shorter periods in that abode, so far as the conditions of their imprisonment are concerned. The position of first-class misdemeanants contains but one real element of punishment, and that is the loss of liberty. Even that element of trial was found by a certain busy journalist—the well-known editor of the *Review of Reviews*—to be in reality a very great boon. He has given an interesting account of his experience during a three months' imprisonment as a first-class misdemeanant, and he states that he never enjoyed himself so much in his life as during that period of detention. His family came to see him, and any intimate friend he chose to admit; but he was free from all unwelcome visitors, and from the constant interruptions and worrying business which falls to the lot of editors and authors. It may be said as regards the so-called raiders that, as soldiers and men of enterprise, the enforced inaction and monotony of daily prison life must be very trying. It may be so, but, regarded as a legal sentence of punishment, it must be admitted that it is of a very mild description.

